# Molecular epidemiology of carbapenemase-producing *Enterobacterales* in Finland, 2012–2018

**DOI:** 10.1007/s10096-020-03885-w

**Published:** 2020-04-19

**Authors:** Kati Räisänen, Outi Lyytikäinen, Jari Kauranen, Eveliina Tarkka, Benita Forsblom-Helander, Juha O. Grönroos, Risto Vuento, Dinah Arifulla, Emmi Sarvikivi, Saija Toura, Jari Jalava

**Affiliations:** 1 Department of Health Security, Finnish Institute for Health and Welfare, Helsinki, Finland; 2 NordLab, Oulu, Finland; 3 Clinical Microbiology, University of Helsinki, Helsinki, Finland; 4 Helsinki University Hospital, Helsinki, Finland; 5 Department of Clinical Microbiology, Turku University Hospital, Turku, Finland; 6 Department of Microbiology, Fimlab Laboratories Ltd., Tampere, Finland

**Keywords:** CPE, *Enterobacterales*, Finland, Whole genome sequencing, Molecular epidemiology

## Abstract

Carbapenemase-producing *Enterobacterales* (CPE) pose an increasing threat to patient safety and healthcare systems globally. We present molecular epidemiology of CPE in Finland during 2012–2018 with detailed characteristics of CPE strains causing clusters during the same time period. All Finnish clinical microbiology laboratories send *Enterobacterales* isolates with reduced susceptibility to carbapenems or isolates producing carbapenemase to the reference laboratory for further characterization by whole genome sequencing (WGS). In total, 231 CPE strains from 202 patients were identified during 2012–2018. Of the strains, 59% were found by screening and 32% from clinical specimens, the latter were most commonly urine. Travel and/or hospitalization history abroad was reported for 108/171 strains (63%). The most common species were *Klebsiella pneumoniae* (45%), *Escherichia coli* (40%), and *Citrobacter freundii* (6%), and the most common carbapenemase genes *bla*_NDM-like_ (35%), *bla*_OXA-48-like_ (33%), and *bla*_KPC-like_ (31%). During 2012–2018, the annual number of CPE strains increased from 9 to 70 and different sequence types from 7 to 33, and *bla*_OXA-48-like_ genes became the most prevalent. Of the clusters, 3/8 were linked to traveling or hospitalization abroad and 5/8 were caused by *K. pneumoniae* clone clonal complex 258. Most of the clusters were caused by *K. pneumoniae* producing KPC. High variety among different sequence types indicates that majority of CPE cases detected in Finland are likely imported from foreign countries. Nearly one-third of the cases are not found by screening suggesting that there is hidden transmission occurring in the healthcare settings.

**Electronic supplementary material:**

The online version of this article (10.1007/s10096-020-03885-w) contains supplementary material, which is available to authorized users.

## Introduction

Carbapenem-resistant *Enterobacterales* are one of the most significant increasing health threats globally according to the WHO [1]. In addition to carbapenemase genes, carbapenem-producing *Enterobacterales* (CPE) have typically collected other resistance genes and are often extensively drug-resistant or even pan-drug resistant, limiting treatment options [2] and leading subsequently to high fatality [3]. Transmission of CPE primarily occurs in hospitals and other healthcare facilities [4]. Transmission of the *K. pneumoniae* clonal complex (CC) 258 including, sequence types (ST) 258, 11, 340, and 512, has been shown to occur in European, US, and Israeli hospitals and long-term care facilities [5].

The CPE situation varies dramatically in different parts of the world, and also between European countries, from sporadic occurrence to endemic situation [6, 7]. Regions and countries are known to be endemic for a certain carbapenemase, for instance *Klebsiella pneumoniae* carbapenemase (KPC) in the USA, Puerto Rico, Colombia, Brazil, Argentina, Greece, and Italy, New Delhi metallo-β-lactamase (NDM) in Indian subcontinent, China, Bangladesh, and Pakistan, and oxacillinase-48 (OXA-48) in Morocco, Turkey, and Malta [7].

CPE has been rare in Finland, as in the other Nordic countries [8]. Until 2013, the occurrence of CPE in Finland was sporadic and related to traveling, and transmission of CPE between two patients was suspected only once [9]. The first outbreak of colonization with KPC-producing *K. pneumoniae* (KPC-KP) strain ST512 affected nine patients in a primary care hospital in 2013 [10]. A regional outbreak, in which a KPC-KP ST512 strain spread from one hospital to four other healthcare facilities occurred during 2013–2018 and caused several clinical infections [11].

In this report, we present molecular epidemiology of CPE in Finland during 2012–2018 and detailed characterization of CPE strains causing clusters during the same time period.

## Materials and methods

### Surveillance and bacterial strains

In 2010, Finnish Institute for Health and Welfare (THL) gave guidelines to clinical microbiology laboratories to send CPE or carbapenem-resistant isolates for further characterization. From 2016, on CPE surveillance in Finland is based on communicable diseases act (1227/2016). All clinical microbiology laboratories electronically notify *E. cloacae*, *E. coli*, and *K. pneumoniae* isolates with reduced susceptibility to carbapenems to the National Infectious Disease Registry and send bacterial strains with carbapenemase gene to the Expert Microbiology Unit of the THL. Clinical laboratories also send other CPE species for further characterization. In addition to the patient’s identity information and demographics, the laboratories are requested to report specimen type (screening or clinical) and information on the patient’s preceding travel or hospitalization history abroad if known. According to the national guidelines for controlling multidrug-resistant (MDR) microbes, patients who have been hospitalized abroad in the preceding 12 months are placed on contact precautions upon admission and are screened for MDR bacteria, including CPE [12]. For this work, one isolate per patient per species per year was included, multiple isolates if the patient had isolates with different carbapenemase genes or sequence types (ST). Data on preceding hospitalization history abroad were completed from the local infection control nurses. Since 2015, all CPE isolates have routinely been sequenced using whole genome sequencing (WGS) and for this work, the older isolates (from years 2012 to 2014) were sequenced retrospectively.

### Phenotypic and molecular analysis

The species identification was performed in clinical laboratories by matrix-assisted laser desorption/ionization time-of-flight (MALDI-TOF) mass spectrometry (VITEK MS, bioMeriéux, Marcy-L’Etoile, France or Bruker Biotyper, Becton, Dickinson and Company, New Jersey, USA), and antimicrobial susceptibilities were assessed by disk diffusion method or by gradient minimum inhibitory concentration (MIC) determination test (Etest, bioMeriéux, Marcy-L’Etoile, France) and interpreted according to clinical breakpoints as published by the European Committee on Antimicrobial Susceptibility Testing (EUCAST) versions 2.0-8.1, 2012-2018 [13]. Clinical laboratories use multiplex real-time PCR or other molecular amplification techniques for confirmation of carbapenemase genes in isolates with reduced susceptibility to any carbapenem, as directed in The Finnish national guideline [12]. National guideline screening breakpoints for carbapenems are the same for *E. coli*, *E. cloacae*, and *K. pneumoniae* as published in the EUCAST guideline [14] except for meropenem for which screening breakpoint is > 0.12 mg/L (zone diameter < 25 mm). For other *Enterobacterales*, the breakpoints are the same as the EUCAST clinical breakpoints, ≥ 2 mg/L (zone diameter ≤ 22 mm).

Several different commercial or in-house molecular amplification techniques have been used during the study period. However, as based on the national guideline, these methods detect at least *bla*_KPC_, *bla*_NDM_, *bla*_OXA-48_, and Verona integron-encoded metallo-β-lactamase (*bla*_VIM_) genes [12].

WGS was performed on a MiSeq instrument (Illumina, San Diego, CA, USA) as previously described [11]. Analysis with Trimmomatic (version 0.33), fastQC (version 0.11.6), SRST2 version 0.2.0, and SeqSphere+ (Ridom GmbH, Münster, Germany) was accomplished as previously described [11]. Core genome (cg) MLST was performed to *K. pneumoniae* and *E. coli* using available cgMLST scheme from Ridom and *C. freundii* and *K. oxytoca* cgMLST schemes were made in house having 2007 and 2947 targets, respectively. A cut-off of 10 allele differences was used to define clusters in cgMLST analysis. This cut-off has been experimentally determined and used in similar studies previously [11, 15]. WGS data from each of the eight clusters are available from GenBank (Table 1).

**Table 1:** Clusters caused by carbapenemase-producing *Enterobacterales* in Finland, 2012–2018

						Specimen type			
Year(s)	No. of strains	No. of patients	Species	Gene	Sequence type	Screening	Clinical	Link abroad, country	GenBank accession number of strain belonging to cluster
2013	9	9	*K. pneumoniae*	*bla*_KPC-3_	ST512	8	1	No	SAMN14411195
2013–2018	26	23	*K. pneumoniae*	*bla*_KPC-3_	ST512	4	10	No	SAMN14411196
2015–2016	2	2	*K. pneumoniae*	*bla*_KPC-3_	ST512	1	0	Hospital transfer, Italy	SAMN14411197
2016–2018	8	8	*C. freundii*	*bla*_KPC-2_	ST18	2	6	No	SAMN14411198
2016–2018	3	3	*K. pneumoniae*	*bla*_KPC-3_	ST11/NF*	2	1	Yes, Colombia	SAMN14411199
2018	2	2	*K. pneumoniae*	*bla*_OXA-48_	ST395	1	1	Hospitalization, Russia	SAMN14411200
2018	2	2	*K. pneumoniae*	*bla*_OXA-48_	ST307	2	0	No	SAMN14411201
2018	2	2	*K. pneumoniae*	*bla*_OXA-48_	ST273	1	1	No	SAMN14411202

*For one strain completely identical ST was not found, there was one allele difference to ST11

## Results

In total, 231 CPE strains obtained from 202 patients during 2012–2018 were included in the study: 57% (115/202) were from males and the median age of the patients was 56 years (range, 6 months–98 years). Of the strains, 59% (137/231) were found by screening, 32% (74/231) from clinical specimens, and for 9% (20/231) the information was not available. Of the clinical specimens, 42 (57%) were from urine, 8 (11%) from wound or abscess, 7 (9%) from blood, and 5 (7%) from respiratory tract. Travel or hospitalization abroad was reported in 91 patients and travel data was not available for 53 patients, 58 patients had no travel or hospitalization abroad. The most common countries were India (*n* = 29 strains), Greece (*n* = 11), Thailand (*n* = 9), Spain (*n* = 7), and Turkey (*n* = 7). A total of 52 strains were imported after patient had been hospitalized abroad: 10 in India, 7 in Greece, 5 in Egypt, 4 in Spain, 3 in Turkey, 3 in Russia, 3 more in Europe, 16 outside Europe, and in one case, the country was unknown (Electronic Supplementary Material Table 1).

**Fig. 1:**
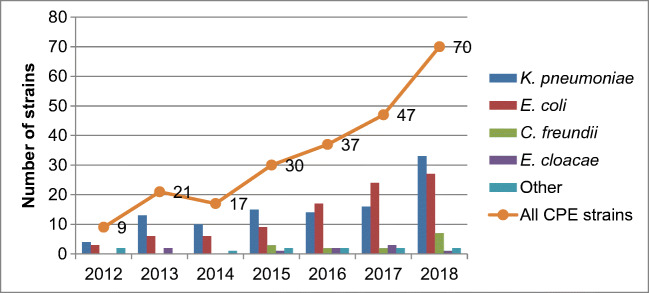
Annual number of carbapenemase-producing *Enterobacterales* species in Finland, 2012–2018

**Fig. 2:**
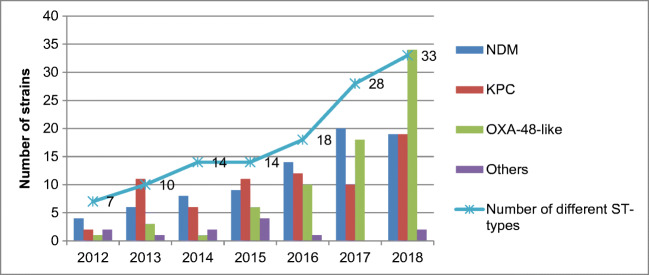
The annual number of sequence types and strains with different carbapenemases of carbapenemase-producing *Enterobacterales* in Finland, 2012–2018

The annual number of CPE strains increased from 9 in 2012 to 70 in 2018 (Fig. 1), and the number of different STs increased from 7 in 2012 to 33 in 2018 (Fig. 2). *K. pneumoniae* was the dominant species during the study period, except for years 2016 and 2017 when *E. coli* was most common. *E. cloacae* was found consistently during the study period, and *C. freundii* only during the last 4 years (2015–2018).

The most common CPE species were *K. pneumoniae* (45%, 105/231), *E. coli* (40%, 92/231), *C. freundii* (6%, 14/231), and *E. cloacae* (4%, 9/231) (Table 1). Most prevalent carbapenemase genes were *bla*_NDM-like_ (35%, 80/231), *bla*_OXA-48-like_ (33%, 76/231), and *bla*_KPC-like_ (31%, 71/231). Species where carbapenemase gene *bla*_NDM-like_ was detected commonly were *E. coli* (*n* = 50) and *K. pneumoniae* (*n* = 24), *bla*_OXA-48-like_ in *E. coli* (*n* = 39) and *K. pneumoniae* (*n* = 29), and *bla*_KPC-like_ in *K. pneumoniae* (*n* = 55) and *C. freundii* (*n* = 9). Of the individual carbapenemase genes, *bla*_KPC-3_ was the most prevalent followed by *bla*_OXA-48_, *bla*_NDM-5_, and *bla*_NDM-1_, respectively, and these were found in several different species and STs. No plasmid mediated colistin resistance genes were found.

*K. pneumoniae* had 23 different STs among 105 strains and *E. coli* had 37 different STs among 92 strains. Among *K. pneumoniae*, the most prevalent STs were ST512 (*n* = 39), ST258 (*n* = 8), ST11 (*n* = 7), and ST395 (*n* = 7) and among *E. coli*, ST167 (*n* = 11), ST38 (*n* = 9), and ST405 (*n* = 9).

When the patient had travel or hospitalization history in a European region, the most common carbapenemase genes belonged to *bla*_OXA-48-like_ (18/37), in South-East Asia, Western Pacific, and Africa regions, it belonged to *bla*_NDM-like_ (34/40, 3/5, and 2/3 respectively), in Eastern Mediterranean region, it belonged to *bla*_OXA-48-like_ or *bla*_NDM-like_ (10/16 and 7/16 respectively), and in Americas, it belonged to *bla*_KPC-like_ (3/4).

Eight CPE clusters were detected during 2012–2018 (Table 1). The defined cut-off (10 allele differences) was used except in one case: one *C. freundii* isolate with 14 allele differences was defined belonging to the cluster since the patients were hospitalized in the same healthcare facility less than 8 months apart. Five of eight clusters were caused by *K. pneumoniae* strains belonging to the CC258. *K. pneumoniae* ST512 with *bla*_KPC-3_ gene caused three clusters, two large ones with 9 and 23 patients, respectively and one small with two patients. *C. freundii* ST18 with *bla*_KPC-2_ gene caused one cluster with 8 patients and *K. pneumoniae* ST11 with *bla*_KPC-3_ gene one cluster with three patients. Furthermore, there have been three small clusters caused by *K. pneumoniae* strain having *bla*_OXA-48_ gene with different STs. In two clusters, CPE was found more often in clinical than in screening specimens and in four clusters, the first specimen was obtained on clinical basis. In three clusters, the link abroad was identified.

## Discussion

Our study showed that CPE isolates are increasingly found in Finland and have caused several clusters and outbreaks during 2013–2018. The first *K. pneumoniae* producing KPC-2 strain was detected in Finland already in 2009 [16], and thereafter KPC has become one of the most dominant carbapenemases in Finland. KPC was common in *K. pneumoniae* and rare in *E. coli* in our material which is in line with data reported from Europe [8]. *K. pneumoniae* strains belonging to the clonal complex (CC) 258 are wide spread [5], and it has been shown that carbapenemase-producing *K. pneumoniae* strains are more eager to spread than other strains [4]. Noteworthy, despite that *E. coli* was the most prevalent species during 2 years of the study period and the second prevalent during the other years, no carbapenemase-producing *E. coli* clusters were detected in Finland. MLST results also showed that *K. pneumoniae* had fewer STs than *E. coli*, indicating more clonal population structure.

High number of *C. freundii* detected in our study was related to the cluster with eight cases. Germany has described one KPC-2 producing *C. freundii* outbreak with 6 cases [17]. These outbreaks indicate that also *C. freundii* with KPC can cause outbreaks and be a reservoir of CPE-genes.

We focused only on CPEs, we did not analyse carbapenem-resistant *Enterobacterales* (CRE). It is possible that there were CRE strains with carbapenemase genes that clinical laboratories did not detect by the molecular methods they had in use. However, based on the previous studies in Finland and other European countries, other carbapenemase genes than those recommended for testing in the national guideline are rare [9, 18]. It is also possible that there were *Enterobacterales* strains with carbapenemase genes which had MICs or inhibition zones for carbapenem antibiotic below screening breakpoints. Especially OXA-48-like enzymes are weak carbapenemases and the strains having *bla*_OXA-48-like_ genes can have very low carbapenem MICs. However, also these kinds of strains seem to be very rare [8, 19, 20].

Simultaneously, as the number of strains increased annually, the number of different sequence types increased indicating that importation from different foreign countries played a crucial role in changing molecular epidemiology of CPE. Endemicity of certain CPE-genes can be seen in our material when examining travel destinations and hospitalization abroad in relation to genes imported. Also, wide selection of species, sequence types, and different CPE-genes detected support the hypothesis that most cases are imported, although direct links could not be found for all patients. The history of traveling abroad is not systematically collected for all patients admitted to Finnish healthcare facilities and this information was missing for 53/202 patients. We cannot exclude possible horizontal gene transfer between species, even though it seems more improbable than clonal spread [4].

However, a worrisome phenomenon is that CPE was initially often found from clinical specimens and from patients without direct link abroad indicating hidden local transmissions. This means that there are unknown CPE cases and possible environmental sources challenging CPE control measures. Alarming was also that after detecting a cluster and in spite of infection control measures, onward transmission was not always successfully controlled. One reason for this might be that several *Enterobacterales* are known to survive for long periods in the hospital environment [21]. Therefore, we are currently updating national MDR control guidelines concerning CPE, terminal cleaning, and screening strategies.

In conclusion, CPE findings in Finland are increasing but still the majority are sporadic. The most common CPE so far was *K. pneumoniae* with KPC-3 gene which caused most of the CPE cases in outbreaks. Our CPE findings are similar to those reported by other Nordic countries [22, 23].

## Electronic supplementary material

ESM 1 (XLSX 24 kb)

## Data Availability

The datasets generated during and/or analysed during the current study are available from the corresponding author on reasonable request.

## References

[1] WHO. Antimicrobial resistance, (2017), www.who.int/en/news-room/fact-sheets/detail/antimicrobial-resistance

[2] Perez F, Chakhtoura NGE, Papp-Wallace KM, Wilson BM, Bonomo RA (2016). Treatment options for infections caused by carbapenem-resistant Enterobacteriaceae: can we apply “precision medicine” to antimicrobial chemotherapy?. Expert Opin Pharmacother.

[3] Falagas ME, Lourida P, Poulikakos P, Rafailidis PI, Tansarli GS (2014). Antibiotic treatment of infections due to carbapenem-resistant Enterobacteriaceae: systematic evaluation of the available evidence. Antimicrob Agents Chemother.

[4] David S, Reuter S, Harris SR, Glasner C, Feltwell T, Argimon S, Abudahab K, Goater R, Giani T, Errico G, Aspbury M, Sjunnebo S, Feil EJ, Rossolini GM, Aanensen DM, Grundmann H (2019) Epidemic of carbapenem-resistant Klebsiella pneumoniae in Europe is driven by nosocomial spread. Nat Microbiol. 10.1038/s41564-019-0492-8 10.1038/s41564-019-0492-8 PMC7244338 31358985

[5] Pitout JDD, Nordmann P, Poirel L (2015). Carbapenemase-producing Klebsiella pneumoniae, a key pathogen set for global nosocomial dominance. Antimicrob Agents Chemother.

[6] Brolund A, Lagerqvist N, Byfors S, Struelens MJ, Monnet DL, Albiger B, Kohlenberg A (2019) Worsening epidemiological situation of carbapenemase-producing Enterobacteriaceae in Europe, assessment by national experts from 37 countries, July 2018. Euro Surveill 24. 10.2807/1560-7917.ES.2019.24.9.1900123 10.2807/1560-7917.ES.2019.24.9.1900123 PMC6402177 30862330

[7] Logan LK WR (2017) The epidemiology of carbapenem-resistant Enterobacteriaceae: the impact and evolution of a global menace. J Infect Dis 215:28–36. doi: 10.1093/infdis/jiw282 10.1093/infdis/jiw282 PMC5853342 28375512

[8] Grundmann H, Glasner C, Albiger B (2016). Occurrence of carbapenemase-producing Klebsiella pneumoniae and Escherichia coli in the European survey of carbapenemase-producing Enterobacteriaceae (EuSCAPE): a prospective, multinational study. Lancet Infect Dis.

[9] Österblad M, Kirveskari J, Hakanen AJ, Tissari P, Vaara M, Jalava J (2012). Carbapenemase-producing Enterobacteriaceae in Finland: the first years (2008-11). J Antimicrob Chemother.

[10] Kanerva M, Skogberg K, Ryynänen K, Pahkamäki A, Jalava J, Ollgren J, Tarkka E, Lyytikäinen O (2015). Coincidental detection of the first outbreak of carbapenemase-producing Klebsiella pneumoniae colonisation in a primary care hospital, Finland, 2013. Euro Surveill.

[11] van Beek J, Räisänen K, Broas M et al (2019) Tracing local and regional clusters of carbapenemase- producing Klebsiella pneumoniae ST512 with whole genome sequencing, Finland, 2013 to 2018. Euro Surveill 24. 10.2807/1560-7917.ES.2019.24.38.1800522 10.2807/1560-7917.ES.2019.24.38.1800522 PMC6761573 31552821

[12] Kolho E, Lyytikäinen O, Jalava J Ohje moniresistenttien mikrobien tartunnantorjunnasta. [National guideline for control of multidrug-resistant microbes]. Helsinki: National Institute for Health and Welfare (THL); 2017. Finnish. In: http://urn.fi/URN:ISBN:978-952-302-943-9

[13] The European Committee on Antimicrobial Susceptibility Testing. Breakpoint tables for interpretation of MICs and zone diameters. Version 2.0–8.1, 2012–2018. https://www.eucast.org

[14] Giske GC, Martinez-Martinez L, Cantón R, Stefani S, Skov R, Glupczynski Y, Nordmann P, Wootton M, Miriagou V, Simonsen SG, Zemlickova H, Cohen-Stuart J, Gniadkowski M (2017) EUCAST guidelines for detection of resistance mechanisms and specific resistances of clinical and/or epidemiological importance

[15] Kluytmans-van den Bergh MFQ, Rossen JWA, Bruijning-Verhagen PCJ, Bonten MJM, Frieich AW, Vandenbroucke-Grauls CMJE, Willems RJL, Kluytmans JAJW (2016). Whole-genome multilocus sequence typing of extended-spectrum-beta-lactamase-producing Enterobacteriaceae. J Clin Microbiol.

[16] Österblad M, Kirveskari J, Koskela S, Tissari P, Vuorenoja K, Hakanen AJ, Vaara M, Jalava J (2009) First isolations of KPC-2-carrying ST258 Klebsiella pneumoniae strains in Finland, June and August 2009. Euro Surveill 14 19822122

[17] Schweizer C, Bischoff P, Bender J, Kola A, Gastmeier P, Hummel M, Klefisch F, Schoenrath F, Frühauf A, Pfeifer Y (2019). Plasmid-mediated transmission of KPC-2 carbapenemase in Enterobacteriaceae in critically ill patients. Front Microbiol.

[18] Trepanier P, Mallard K, Meunier D (2017). Carbapenemase-producing Enterobacteriaceae in the UK: a national study (EuSCAPE-UK) on prevalence, incidence, laboratory detection methods and infection control measures. J Antimicrob Chemother.

[19] Woodford N, Xu-McCrae L, Mushtaq S, Wu HHT, Ellington MJ, Lancaster O, Davies F, Donaldson H, Rao GG, Verma A, Wareham DW, Ciesielczuk H, Stone GG, Irani PM, Bracher S, Hawkey PM (2018). Prevalence of carbapenem resistance and carbapenemase production among Enterobacteriaceae isolated from urine in the UK: results of the UK infection-Carbapenem Resistance Evaluation Surveillance Trial (iCREST-UK). J Antimicrob Chemother.

[20] Fattouh R, Tijet N, McGeer A, Poutanen SM, Melano RG, Patel SN (2015). What is the appropriate meropenem MIC for screening of carbapenemase-producing Enterobacteriaceae in low-prevalence settings?. Antimicrob Agents Chemother.

[21] Smismans A, Ho E, Daniels D, Ombelet S, Mellaerts B, Obbels D, Valgaeren H, Goovaerts A, Huybrechts E, Montag I, Frans J (2019). New environmental reservoir of CPE in hospitals. Lancet Infect Dis.

[22] Löfmark S, Sjöström K, Mäkitalo B, Edquist P, Wisell KT, Giske CG (2015). Carbapenemase-producing Enterobacteriaceae in Sweden 2007–2013: experiences from seven years of systematic surveillance and mandatory reporting. Drug Resist Updat.

[23] Samuelsen Ø, Overballe-Petersen S, Bjørnholt JV, Brisse S, Doumith M, Woodford N, Hopkins KL, Aasnæs B, Haldorsen B, Sundsfjord A, on behalf of The Norwegian Study Group on, CPE (2017). Molecular and epidemiological characterization of carbapenemase-producing Enterobacteriaceae in Norway, 2007 to 2014. PLoS One.

